# Genetic, epigenetic, and environmental factors controlling oxytocin receptor gene expression

**DOI:** 10.1186/s13148-021-01017-5

**Published:** 2021-01-30

**Authors:** Joshua S. Danoff, Kelly L. Wroblewski, Andrew J. Graves, Graham C. Quinn, Allison M. Perkeybile, William M. Kenkel, Travis S. Lillard, Hardik I. Parikh, Hudson F. Golino, Simon G. Gregory, C. Sue Carter, Karen L. Bales, Jessica J. Connelly

**Affiliations:** 1 Department of Psychology, University of Virginia, 102 Gilmer Hall, P.O. Box 400400, Charlottesville, VA 22904 USA; 2 The Kinsey Institute, Indiana University, 150 S Woodlawn Avenue, Bloomington, IN 47405 USA; 3 Department of Psychological and Brain Sciences, University of Delaware, 105 The Green, Newark, DE 19716 USA; 4 Division of Infectious Diseases and International Health, University of Virginia, 345 Crispell Drive, Charlottesville, VA 22908 USA; 5 Research Computing, University of Virginia, 560 Ray C. Hunt Drive, Charlottesville, VA 22903 USA; 6 Duke Molecular Physiology Institute, Duke University School of Medicine, 300 N Duke St, Durham, NC 27701 USA; 7 Department of Psychology, University of California, Davis, One Shields Avenue, Davis, CA 95616 USA

**Keywords:** Oxytocin receptor, DNA methylation, Early life experience, Prairie vole

## Abstract

**Background:**

The neuropeptide oxytocin regulates mammalian social behavior. Disruptions in oxytocin signaling are a feature of many psychopathologies. One commonly studied biomarker for oxytocin involvement in psychiatric diseases is DNA methylation at the oxytocin receptor gene (*OXTR*). Such studies focus on DNA methylation in two regions of *OXTR*, exon 3 and a region termed MT2 which overlaps exon 1 and intron 1. However, the relative contribution of exon 3 and MT2 in regulating *OXTR* gene expression in the brain is currently unknown.

**Results:**

Here, we use the prairie vole as a translational animal model to investigate genetic, epigenetic, and environmental factors affecting *Oxtr* gene expression in a region of the brain that has been shown to drive *Oxtr* related behavior in the vole, the nucleus accumbens. We show that the genetic structure of *Oxtr* in prairie voles resembles human *OXTR*. We then studied the effects of early life experience on DNA methylation in two regions of a CpG island surrounding the *Oxtr* promoter: MT2 and exon 3. We show that early nurture in the form of parental care results in DNA hypomethylation of *Oxtr* in both MT2 and exon 3, but only DNA methylation in MT2 is associated with *Oxtr* gene expression. Network analyses indicate that CpG sites in the 3′ portion of MT2 are most highly associated with *Oxtr* gene expression. We also identify two novel SNPs in exon 3 of *Oxtr* in prairie voles and a novel alternative transcript originating from the third intron of the gene. Expression of the novel alternative transcript is associated with genotype at SNP KLW2.

**Conclusions:**

These results identify putative regulatory features of *Oxtr* in prairie voles which inform future studies examining *OXTR* in human social behaviors and disorders. These studies indicate that in prairie voles, DNA methylation in MT2, particularly in the 3′ portion, is more predictive of *Oxtr* gene expression than DNA methylation in exon 3. Similarly, in human temporal cortex, we find that DNA methylation in the 3′ portion of MT2 is associated with *OXTR* expression. Together, these results suggest that among the CpG sites studied, DNA methylation of MT2 may be the most reliable indicator of *OXTR* gene expression. We also identify novel features of prairie vole *Oxtr*, including SNPs and an alternative transcript, which further develop the prairie vole as a translational model for studies of *OXTR*.

## Background

A major goal of translational neuroscience is to identify biomarkers of psychiatric disorders which can be used to inform diagnosis, predict possible courses of disease progression, or predict likelihood of response to a particular treatment [1]. A common target of efforts to identify biomarkers of psychiatric disorders is the oxytocin system [2]. The oxytocin system is an attractive target for biomarkers because of its involvement in many psychiatric disorders [3], modulation of a wide range of physiological processes [4], and potential use as medication via intranasal administration [5–7]. While many studies use serum oxytocin levels as a biomarker, proper collection and measurement of oxytocin is challenging, and interpretation may vary based on methodology [8].

Epigenetic modifications are promising biomarkers for disease since they are easily measured, variable in the population, and can be influenced by the environment [9]. A prominent epigenetic biomarker is DNA methylation, which is the addition of a methyl group to cytosine residues. DNA methylation typically occurs when cytosine is followed by guanine and generally leads to decreased gene expression [10]. One epigenetic biomarker for the oxytocin system is DNA methylation of the oxytocin receptor gene, *OXTR*. *OXTR* structurally consists of four exons and three introns in humans [11]. DNA methylation in MT2, a 405 bp region overlapping exon 1 and intron 1 of *OXTR*, regulates transcription both in vivo and in vitro [12]. *OXTR* is expressed throughout the human brain, with highest levels of expression in striatum, thalamus, and olfactory regions [13]. Reduced expression of *OXTR* has been documented in the superior temporal gyrus in autism [14] and in the posterior medial temporal cortex in schizophrenia [15]. Increased expression of *OXTR* was reported in the prefrontal cortex of patients with depression and bipolar disorder [16].

Dysregulation of *OXTR* expression in multiple psychopathologies has led many to study the epigenetic state of *OXTR* in both typical and clinical groups. These studies have generally focused on two regions of the *OXTR* gene: MT2 (covering much of exon 1 and some of intron 1) and exon 3. For example, dysregulated DNA methylation in MT2 has been associated with autism [14], callous–unemotional traits [17, 18], anorexia nervosa [19, 20], schizoaffective disorders [21, 22], post-partum depression [23], attachment anxiety [24], obsessive compulsive disorder [25], and anxiety and depression [26]. DNA methylation in MT2 is associated with neural endophenotypes in typical populations: DNA methylation is positively correlated with brain activity during animacy perception [27], emotion perception [28, 29], and social attention [30]. DNA methylation in exon 3 has also been associated with several psychopathologies, including anxiety and depression [26, 31], social anxiety disorder [32], obsessive compulsive disorder [25, 33], post-traumatic stress disorder [34], callous–unemotional traits [17], and social communication deficits [35]. Despite the many studies connecting epigenetic markers at *OXTR* and psychological outcomes, several questions remain. Most studies measure DNA methylation in peripheral tissues such as blood or saliva, and the association between DNA methylation in these tissues and the brain is not established for all CpG sites. Furthermore, even if DNA methylation is measured in the brain, it is not yet known what the relative contributions of DNA methylation in the MT2 region and exon 3 region are in controlling *OXTR* expression.

In order to investigate epigenetic mechanisms controlling *OXTR* expression in the brain we use prairie voles as an animal model. Prairie voles are highly social rodents with characteristics resembling human social behavior including pair bonding and biparental care of offspring [36]. We have previously shown that prairie voles are also a good model for studies of *OXTR*: the region of MT2 containing CpG sites -901, -924, and -934 is highly conserved in prairie voles and humans, but not in mice or rats [37]. We have also shown that DNA methylation in this region is malleable to early life experience, which ultimately leads to changes in gene expression in the juvenile period [37] and adulthood [38]. In this study, we clarify the genetic structure of *Oxtr* in prairie voles and compare the gene structure to human *OXTR*. We then investigate the relationships between DNA methylation in MT2, DNA methylation in exon 3, and *Oxtr* expression in the prairie vole brain. Finally, we describe novel findings related to prairie vole *Oxtr* including previously unidentified SNPs in exon 3 and a novel alternative transcript present in the nucleus accumbens.

## Results

### Oxtr gene structure in prairie voles resembles human OXTR gene structure with high homology in CpG-rich regulatory regions

**Fig. 1:**
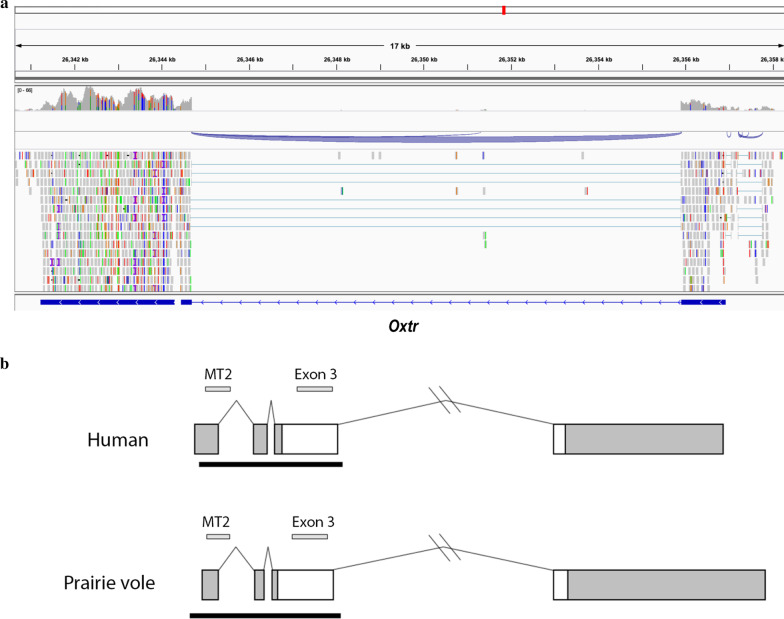
Oxtr gene structure and homology in prairie voles and humans. **a** RNA-seq evidence of *Oxtr* gene structure in prairie voles. The region shown is micOch1.0 scaffold JH996431.1: 26,340,626–26,358,364. **b** Gene schematic of *OXTR* in humans and *Oxtr* in prairie voles. Exons are indicated by boxes (coding, white; untranslated, gray). Introns are shown with solid lines. The black boxes below the transcripts delineate a CpG island identified using the CpG island function of the sequence manipulation suite [40]. Gray boxes above the transcripts denote conserved regions of the CpG island labeled as MT2 and exon 3. These conserved regions were assayed for DNA methylation

Previously, the structure of the *Oxtr* gene in prairie voles was reported to have two exons which are homologous to exons 3 and 4 in humans (MicOch1.0, GCA_000317375.1, Ensembl release 100). However, this gene in other rodents has four exons, similar to humans, including two exons at the 5′ end of the gene which are not translated [39]. In order to determine the full structure of *Oxtr* in prairie voles, we completed RNA-sequencing on RNA from the nucleus accumbens. Viewing the read alignment in the Integrated Genomes Viewer (IGV) reveals that this gene has four exons and three introns, much like the human gene (Fig. 1a, b). In addition, the 3′ UTR extends further than previously reported.

We assessed the homology between prairie voles and human for two CpG-rich regions in *OXTR* where many human studies associated DNA methylation and neuropsychological outcomes, MT2 and exon 3. Homology was determined using PRSS (DNA:DNA). For the MT2 region, this analysis identified a conserved region between human and prairie voles with 64.3% shared identity (Additional file 1: Fig. S1A; Smith-Waterman score: 566; 64.3% similar; Z-score: 120.2; bits: 120.2; E(1000): 1 × 10^–31^). For the exon 3 region, this analysis identified a conserved region between human and prairie voles with 88.4% identity (Additional file 1: Fig. S1B; Smith-Waterman score: 2275; 88.4% similar; Z-score: 1966.2; bits: 373.7; E(1000): 1 × 10^–107^).

### DNA methylation in MT2 is sensitive to early life experience and associated with Oxtr gene expression in prairie vole and human brains

**Fig. 2:**
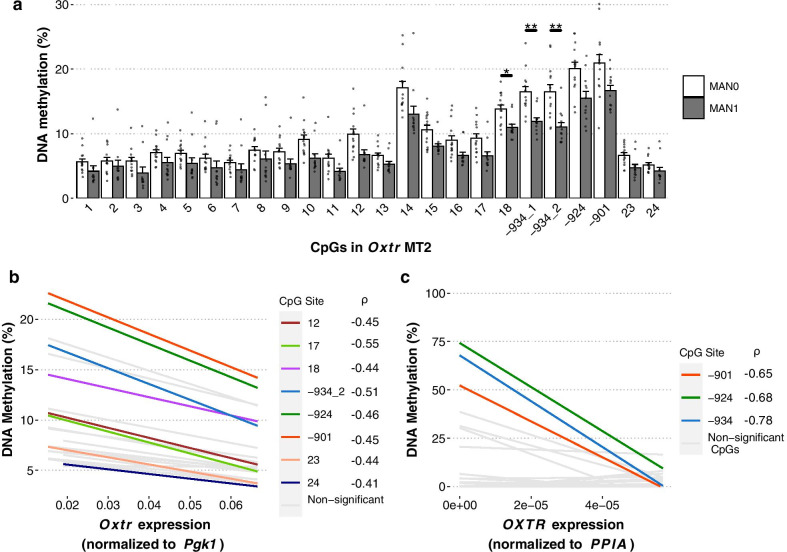
DNA methylation in MT2 is impacted by early life experience and associated with *Oxtr* expression. **a** Offspring that received less parental care (MAN0) have increased DNA methylation across MT2 in the nucleus accumbens (13 male–female sibling pairs; MAN0: *n* = 7 males, 7 females; MAN1: *n* = 6 males, 6 females; mixed effect model with fixed effect of handling condition and random effects of individual and CpG site; Type II Wald F test *F*_(1,24)_ = 8.386, *p* = 0.008). Follow-up t tests between handling conditions at each site were conducted with Bonferroni correction; *p < 0.05, **p < 0.01. **b** DNA methylation in MT2 is associated with *Oxtr* expression in prairie vole nucleus accumbens. Spearman’s rank correlation was used to determine correlation. Sites with significant correlations (not corrected for multiple comparisons) are shown in color while nonsignificant sites are in gray. Spearman’s rho and p values for each site are in Additional file 1: Table S1. **c** DNA methylation in MT2 is associated with *OXTR* expression in human temporal cortex. Spearman’s rank correlation was used to determine correlation. Sites with significant correlations (not corrected for multiple comparisons) are shown in color while nonsignificant sites are in gray. Spearman’s rho and p values for each site are in Additional file 1: Table S2

We have previously shown that early life experience in the form of nurture prevents de novo DNA methylation on four sites in MT2 (-934_1, -934_2, -924, and -901) and leads to increased *Oxtr* gene expression as juveniles [37]. We next aimed to characterize how early life experience affects DNA methylation on other sites in MT2. We used a mixed model analysis to account for correlation between DNA methylation values at these sites (Additional file 1: Fig. S2A). Across all sites in MT2, offspring that received less parental care (MAN0) have increased DNA methylation (Fig. 2a, type II Wald F test *F*_(1,24)_ = 8.386, *p* = 0.008). Several of the sites are significantly correlated with expression of *Oxtr* (Fig. 2b, Additional file 1: Table S1). In order to determine if this model is instructive for studying humans, we compared *OXTR* DNA methylation to gene expression from human samples collected from Brodmann Area 41/42 [14]. In human brain tissue, there is also a significant correlation between DNA methylation at CpG sites -934, -924, and -901 and *OXTR* gene expression (Fig. 2c, Additional file 1: Table S2, -934: *ρ* = − 0.78, *p* = 0.007; − 924: *ρ* = − 0.68, *p* = 0.02; − 901: *ρ* = − 0.66, *p* = 0.028).

### CpG sites -934_1, -934_2, -924, and -901 are most highly associated with Oxtr gene expression

**Fig. 3:**
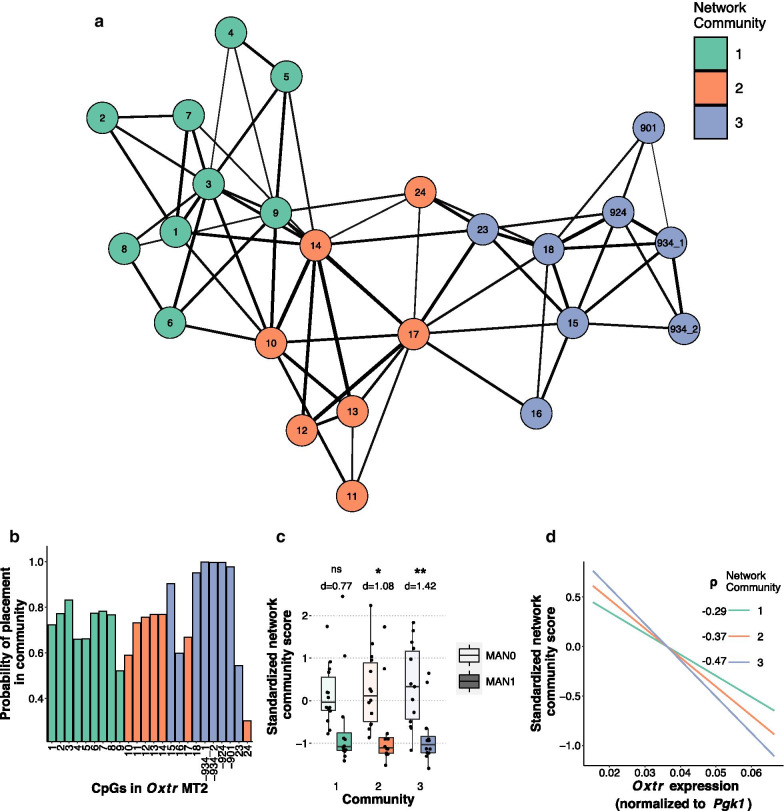
Network analysis of MT2 DNA methylation reveals importance of 3′ region. **a** Community structure network of CpG sites in MT2 containing three communities: community 1 (green) which contains 5′ CpGs, community 2 (orange) which contains mostly CpGs in the middle of MT2, and community 3 (blue) which contains mostly 3′ CpGs. The weight of edges indicates higher covariance between CpG sites. **b** Item stability scores for each CpG site. CpGs with scores greater than 0.8 are considered stable. **c** Animals raised by low care parents (MAN0) have higher standardized network community scores, which represent DNA methylation within the community (13 male–female sibling pairs; MAN0: *n* = 7 males, 7 females; MAN1: *n* = 6 males, 6 females; 2 way handling condition x community ANOVA, main effect of handling condition, *F*_(1, 72)_ = 22.41, *p* = 1.1e-5; no effect of community, *F*_(2, 72)_ = 0.001, *p* = .99. Follow-up t tests between handling conditions at each community were conducted with Bonferroni correction (*p < 0.05, **p < 0.01). Cohen’s d is provided to measure effect size.) The effect size of handling condition on standardized network community score is largest in community 3. **d** Standardized network community scores for each community are correlated with *Oxtr* gene expression using Spearman’s rank correlation; community 1: *ρ* = -0.29, *p* = 0.086; community 1: *ρ* = -0.37, *p* = 0.03; community 3: *ρ* = -0.47, *p* = 0.003

DNA methylation at CpG sites in MT2 are highly correlated with each other (Additional file 1: Fig. S2A). In order to further understand the structure of MT2, we employed exploratory graph analysis (EGA), a dimensionality reduction technique and network structure analysis tool [41, 42]. This tool allows us to cluster CpG sites in an unsupervised manner based on the covariance of DNA methylation values into distinct groups which provide non-redundant information. The results from EGA suggest three distinct communities of CpG sites, largely reflecting the physical structure of MT2: community 1 contains 5′ sites, community 2 contains sites in the middle, and community 3 contains 3′ sites (Fig. 3a, Additional file 1: Fig. S2B-C). The reliability of the community structure was determined using 1000 bootstrapped replications [43]. The probability of each CpG site remaining in their community is shown in Fig. 3b. Probabilities greater than 0.8 are considered stable. Notably, CpG sites -934_1, -934_2, -924, and -901 all remain in their community nearly 100% of the time, and this stability may indicate that these CpG sites are most tightly co-regulated.

In order to determine which community of CpG sites are most sensitive to early nurture, we compared standardized network community scores [44] for each community between the two handling groups. The standardized network community scores are compressed representations of the original DNA methylation data from CpG sites within the community and are analogous to component vectors from principal components analysis. In this way, the network scores represent weighted DNA methylation values from CpG sites within each network community. The standardized network community scores differed by handling condition but not by community (Fig. 3c, two-way community x handling condition ANOVA, main effect of handling condition *F*_(1, 72)_ = 22.41, *p* = 1.1e-5, no effect of community *F*_(2, 72)_ = 0.001, *p* = 0.99). We then performed follow-up t tests to determine which community was most affected by early nurture. The largest effect was found in community 3, which contains sites -934_1, -934_2, -924, and -901 (Welch’s two sample t test with Bonferroni correction and Cohen’s d; community 1: t_(17.3)_ = 1.88, *p* = 0.229, d = 0.77; community 2: t_(22.7)_ = 2.72, *p* = 0.037, d = 1.08; community 3: t_(23.4)_ = 3.70, *p* = 0.003, d = 1.42), indicating that DNA methylation at this cluster of sites is particularly sensitive to early nurture.

We then correlated standardized network community score with *Oxtr* expression. Community 3 most correlates with gene expression (Fig. 3d; community 1: *ρ* = -0.29, *p* = 0.086; community 1: *ρ* = -0.37, *p* = 0.03; community 3: *ρ* = -0.47, *p* = 0.003). Notably, community 3 contains five of the eight sites in MT2 where DNA methylation significantly correlates with *Oxtr* expression (Fig. 2b). This is also the community where DNA methylation is most sensitive to early life experience, indicating that CpG sites in this community may be functionally responsible for regulation of gene expression.

### DNA methylation in exon 3 is sensitive to early life experience but is not associated with gene expression

**Fig. 4:**
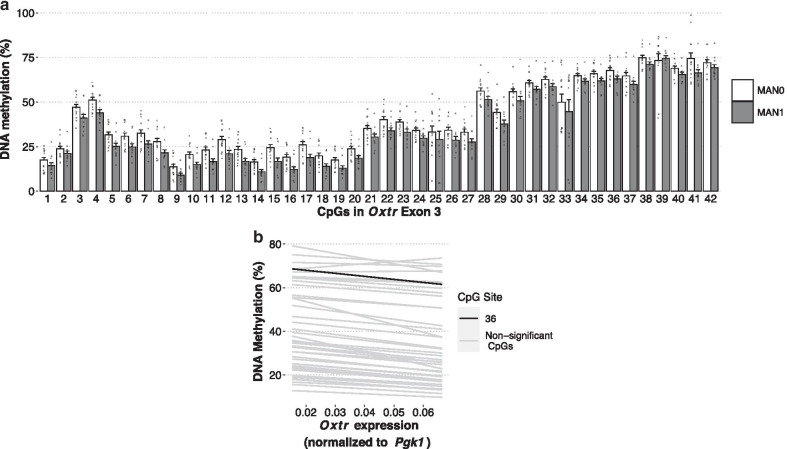
Genetic and epigenetic features of exon 3 do not control *Oxtr* expression. **a** Offspring receiving less parental care (MAN0) have decreased DNA methylation across exon 3 in the nucleus accumbens (13 male–female sibling pairs; MAN0: *n* = 7 males, 7 females; MAN1: *n* = 6 males, 6 females; mixed effect model with fixed effect of handling condition and random effects of individual and CpG site; Type II Wald F test *F*_(1,24)_ = 8.386, *p* = 0.018. CpG sites are numbered according to Additional file 1: Fig S1B. **b** DNA methylation in exon 3 is not robustly associated with *Oxtr* expression in prairie vole nucleus accumbens. Spearman’s rank correlation was used to determine correlation. CpG 36, the only site that has a significant correlation with *Oxtr* expression (not corrected for multiple comparisons) is shown in black. Sites that do not have a significant correlation with *Oxtr* expression are in gray. Spearman’s rho and p values for each site are in Additional file 1: Table S3

**Fig. 5:**
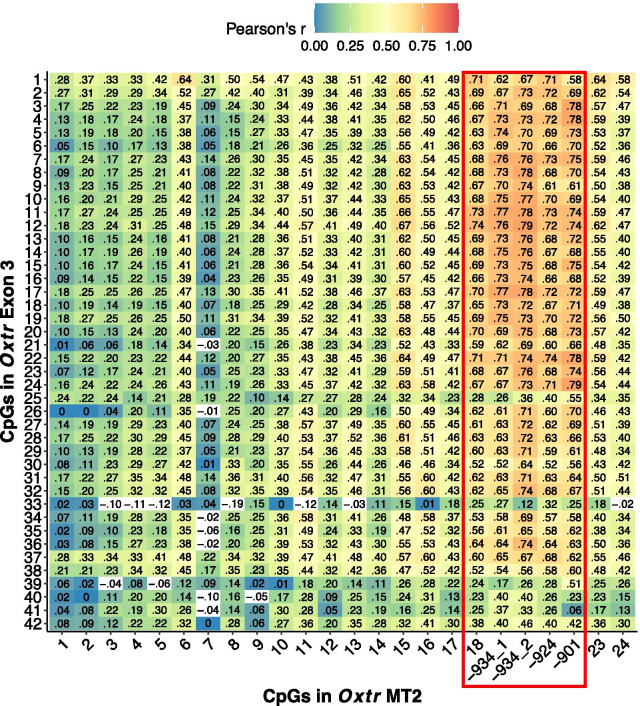
Correlation of DNA methylation in MT2 CpG sites and exon 3 CpG sites. Pearson’s R correlation of DNA methylation in MT2 and exon 3 CpG sites. The red box around MT2 CpGs 18 through -901 indicates that these CpGs have the highest correlation with exon 3 sites

In human studies, DNA methylation at CpG sites within exon 3 of *Oxtr* have been associated with several neuropsychological outcomes [17, 26, 31–35]. In prairie voles there is a highly conserved region within exon 3 that contains 42 CpG sites (Fig. 1b). We analyzed DNA methylation across this region and found that DNA methylation in exon 3 is sensitive to early life experience (Fig. 4a, fixed main effect of handling condition, type II Wald F test *F*_(1,24)_ = 8.386). Follow-up t tests were completed at each site to determine the sites most sensitive to early life experience, but no sites remained significant after Bonferroni correction. DNA methylation in exon 3 was only associated with *Oxtr* gene expression at CpG 36 (Fig. 4b, Additional file 1: Table S3). Thus, while early nurture affects DNA methylation in both MT2 and exon 3 of *Oxtr*, DNA methylation levels in MT2 appear to be an important indicator of expression of the gene but the same is not true of exon 3. Studies in humans identifying associations between DNA methylation and neuropsychological outcomes likely find such results because DNA methylation in exon 3 is highly correlated with DNA methylation in the 3′ portion of MT2 (Fig. 5).

### Identification of two single nucleotide polymorphisms (SNPs) within exon 3 which are not associated with Oxtr gene expression

Within exon 3 there are two CpG sites with patterns of DNA methylation with a cluster of prairie voles with DNA methylation between 50–75%, a cluster between 25–30%, and a few individuals near 0% methylation. We hypothesized that these two sites are polymorphic with one allele preserving the CpG and the other disrupting the CpG such that the cytosine is not methylated. In the same cohort of MAN0 and MAN1 animals, pyrosequencing analysis revealed that these two sites, KLW1 (CpG 25, JH996431.1:26,356,275, micOch1.0) and KLW2 (CpG 33, JH996431.1:26,356,107, micOch1.0), are indeed single nucleotide polymorphisms (SNPs). KLW1 is an A/G SNP where A is the minor allele in this population (frequency = 0.33). KLW2 is also an A/G SNP where A is the minor allele in this population (frequency = 0.27). Both SNPs are in Hardy–Weinberg equilibrium in this population (Haldane’s exact test [45]; KLW1: D = -0.22, *p* = 0.84; KLW2: D = -0.11, *p* = 0.82). We also tested if these SNPs are in linkage disequilibrium with each other or with another SNP in this gene, NT213739 [46]. All three SNPs are in linkage disequilibrium (Table 1).

**Table 1:** Linkage of SNPs KLW1, KLW2, and NT213739

SNP 1	SNP 2	*r*^2^	*χ*^2^	*p* value
KLW1	KLW2	0.18	9.29	0.002
KLW1	NT213739	0.10	5.27	0.022
KLW2	NT213739	0.08	4.00	0.046

**Fig. 6:**
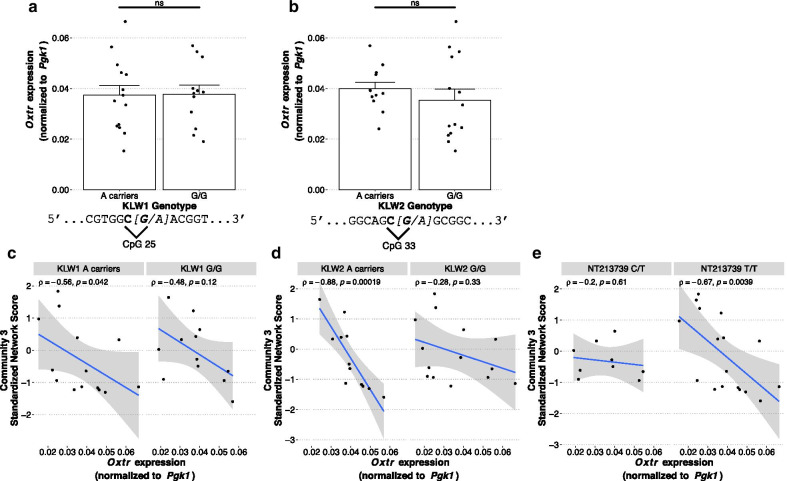
Genetic x epigenetic interactions affecting *Oxtr* expression. **a** Genotype at KLW1 does not impact *Oxtr* expression (A carriers: *n* = 14; G/G: *n* = 12; Welch two sample t test, t_23.9_ = -0.06, *p* = 0.95). Animals are grouped by the presence of minor allele (A) because of low abundance of A/A homozygotes. The DNA sequence surrounding KLW1 is shown in the below graph. **b** Genotype at KLW2 does not impact *Oxtr* expression (A carriers: *n* = 12; G/G: *n* = 14; Welch two sample t test, t_20.4_ = -0.89, *p* = 0.38). Animals are grouped by presence of minor allele (A) because of low abundance of A/A homozygotes. The DNA sequence surrounding KLW2 is shown in the below graph. **c** The relationship between MT2 DNA methylation (represented by community 3 standardized network score) and *Oxtr* expression does not differ between KLW1 genotypes (A carriers: *n* = 14, R = -0.49, *p* = 0.076; G/G: *n* = 12, R = -0.51, *p* = 0.091), but D) does differ between KLW2 genotypes (A carriers: *n* = 12, R = -0.84, *p* = 0.00065; G/G: *n* = 14, R = -0.35, *p* = 0.22) and E) NT213739 genotypes (C/T: *n* = 9, R = -0.17, *p* = 0.66; T/T: *n* = 17, R = -0.61, *p* = 0.01)

However, a larger sample is necessary for more accurate quantitation of linkage disequilibrium. We next examined the effect of genotype at KLW1 and KLW2 on expression of *Oxtr*. Neither KLW1 genotype nor KLW2 genotype impacted *Oxtr* expression (Fig. 6a, b; KLW1: t_(23.9)_ = -0.06, *p* = 0.95; KLW2: t_(20.4)_ = -0.89, *p* = 0.38).

An interaction between DNA methylation of *OXTR* and SNP rs53576 genotype has been reported in relation to neuropsychological outcomes in humans [23, 31, 35]. This led us to hypothesize that there are genetic x epigenetic interactions affecting *Oxtr* expression. In order to study this in the prairie vole sample, we asked if the relationship between the standardized network community score of community 3, which represents DNA methylation at the 3′ end of MT2, and *Oxtr* expression differs by genotype at SNPs KLW1, KLW2, or NT213739. There are no significant interactions, likely because of the small sample size, but interesting patterns emerge. The correlation between community 3 standardized network score and *Oxtr* expression is very similar in KLW1 A carriers and G/G voles (*ρ* = -0.56 and -0.48, respectively; Fig. 6c). ‘A’ carriers (A/A and A/G) of KLW2 have a stronger correlation between these measures compared to KLW2 G/G voles (*ρ* = -0.88 and -0.28, respectively; Fig. 6d). Voles with T/T genotype at NT213739 also have a stronger correlation between these two measures compared to C/T individuals (*ρ* = -0.67 and -0.20, respectively; Fig. 6e). These results suggest a genetic by epigenetic interaction may impact *Oxtr* expression, but further studies with larger samples from several prairie vole colonies are needed to test this hypothesis.

### Identification of an alternative transcript of Oxtr beginning in intron 3 which is associated with KLW2 genotype

**Fig. 7:**
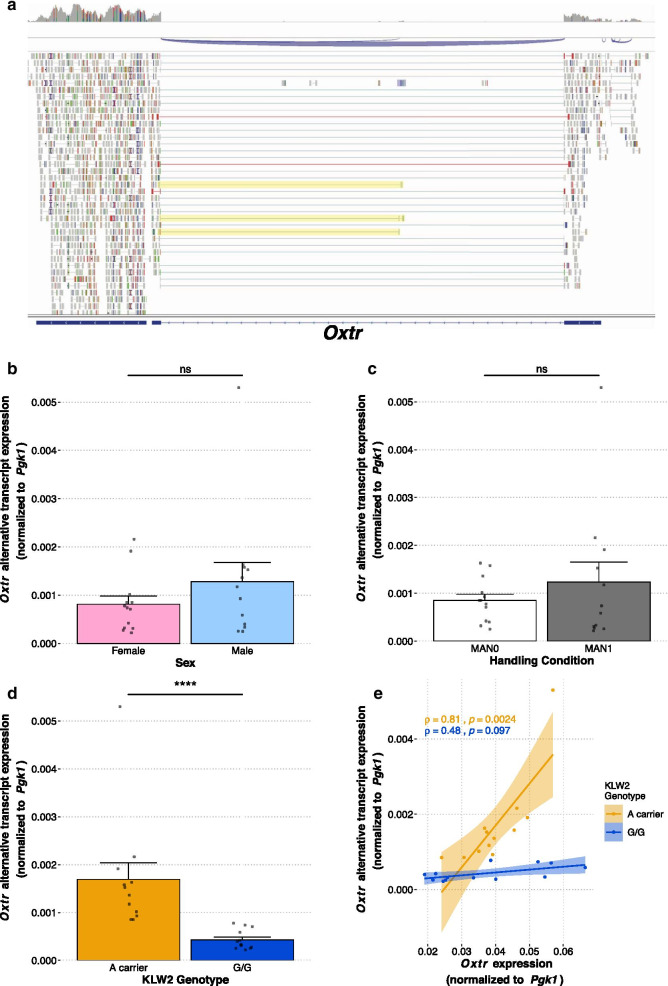
Identification of an alternative transcript of *Oxtr* that is associated with KLW2 genotype. **a** RNA-seq evidence of a novel alternative transcript of *Oxtr* originating in the third intron. Reads providing evidence of this transcript are highlighted in yellow. **b** Expression of the alternative transcript is not impacted by sex (males: *n* = 13, females: *n* = 13; Wilcoxon rank-sum test, *W* = 63, *p* = 0.44). **c** Expression of the alternative transcript is not impacted by handling condition (MAN0: *n* = 14, MAN1: *n* = 12; Wilcoxon rank-sum test, *W* = 82, *p* = 0.85). **d** Expression of the alternative transcript is associated with the A allele of KLW2 (A carrier: *n* = 12, G/G: *n* = 14; Wilcoxon rank sum test, *W* = 156, *p* = 3.85e-7). **e** Expression of the alternative transcript is positively correlated with expression of the main *Oxtr* transcript in animals carrying the A allele of KLW2 (2 way KLW2 genotype x *Oxtr* main transcript expression ANOVA, interaction effect *F*_(1,21)_ = 23.723, *p* = 8.14e-5, adjusted *R*^2^ = 0.734; A carriers: Spearman’s *ρ* = 0.81, *p* = 0.0024; G/G: Spearman’s *ρ* = 0.48, *p* = 0.097)

In addition to *Oxtr* gene structure, RNA-seq data revealed a possible alternative transcript in *Oxtr* consisting of a small exon in the third intron of the main transcript and the fourth exon of the main transcript (Fig. 7a, reads highlighted in yellow). We then measured this transcript in samples from the early nurture experiment using RT-PCR. The transcript is present in all samples, and there are no differences in expression based on nurture (Fig. 7b, W = 82, *p* = 0.85) or sex (Fig. 7c, W = 63, *p* = 0.44). Expression of the alternative transcript is associated with the KLW2 A allele (Fig. 7d, W = 156, *p* = 3.85e-7). Alternative transcript expression is not associated with either KLW1 genotype (Additional file 1: Fig. S3A, *W* = 77, *p* = 0.98) or NT213739 genotype (Additional file 1: Fig. S3B, *W* = 42, *p* = 0.10).

We next asked if the main transcript and this alternative transcript are co-regulated, meaning that animals with high expression of the main transcript could also have high levels of alternative transcript expression. A two-way ANOVA was conducted to determine the influence of main transcript expression while accounting for KLW2 genotype. There was a significant interaction between the expression of the main *Oxtr* transcript and KLW2 genotype (Fig. 7e, F_(1,21)_ = 23.723, *p* = 8.14e-5) such that *Oxtr* main transcript expression is positively correlated with alternative transcript expression in KLW2 A carriers (*ρ* = 0.81, *p* = 0.0024) but there is no correlation in animals with KLW2 G/G genotype (*ρ* = 0.48, *p* = 0.097). Thus, it appears that the main transcript of *Oxtr* is co-regulated with the alternative transcript via a mechanism that is associated with the A allele of KLW2.

## Discussion

The results from these studies clarify the contributions of genetic, epigenetic, and environmental factors in determining *Oxtr* expression in the prairie vole brain. We show that the structure of *Oxtr* in prairie voles resembles human *OXTR*. Within the CpG island spanning the first 3 exons of *OXTR*, two regions have been associated with human neuropsychological outcomes: MT2 and exon 3, both of which are highly conserved in the prairie vole. Using the prairie vole model, we show that *Oxtr* expression in the nucleus accumbens is significantly associated with DNA methylation in MT2 but not DNA methylation in exon 3. Furthermore, network analysis implemented in EGA indicates that DNA methylation in the 3′ end of MT2, which contains CpG sites -901, -924, -934_1, and -934_2 that we have previously reported on [37, 38], is most strongly associated with *Oxtr* expression. Notably, we previously showed that DNA methylation of these CpG sites in whole blood samples are correlated with gene expression in the brain in prairie voles [37]. Additionally, we show that CpG sites -901, -924, and -934 are strongly associated with *OXTR* expression in human temporal cortex while other sites within MT2 are not. Previous studies finding associations between DNA methylation in exon 3 and neuropsychological outcomes in humans likely find this because DNA methylation in exon 3 reports on DNA methylation at CpG sites in the 3′ portion of MT2. Collectively, these results indicate that human studies measuring *OXTR* DNA methylation should focus measurements on MT2, where DNA methylation in both central and peripheral tissues serve as a biomarker for gene expression in the brain.

Our findings expand upon our previous studies examining the relationship between early life experience and *Oxtr* DNA methylation. Here, we show that increased parental care results in hypomethylation across MT2 and exon 3 in the prairie vole brain. These results mirror human studies which find that DNA methylation of *OXTR* is impacted by early life experience in both MT2 [47, 48] and exon 3 [49]. Additionally, childhood abuse in humans interacts with *OXTR* DNA methylation in both MT2 and exon 3 to predict anxiety and depression [26]. Our results further establish prairie voles as an excellent rodent model for studying how early life experience impacts regulation of the oxytocin system and social behavior.

We identified two novel SNPs in prairie vole *Oxtr* within exon 3. Both are A/G SNPs where A is the minor allele and disrupts a CpG site. These SNPs, in addition to the previously identified SNP NT213739 [46], offer an opportunity to model the common association of *OXTR* SNPs with behavioral and psychiatric outcomes in humans [50]. Our results show that neither KLW1 nor KLW2 genotype is associated with *Oxtr* expression in the nucleus accumbens. It was previously reported that the C allele of NT213739 was associated with increased *Oxtr* expression in the prairie vole nucleus accumbens [46], although our group found no association [37]. However, we found that there is a stronger negative association between *Oxtr* DNA methylation and gene expression in animals with A/A and A/G genotypes at KLW2 compared to those with G/G genotypes. Similarly, we found that animals with T/T genotype at NT213739 show a strong negative association between *Oxtr* DNA methylation and gene expression while there was no association in animals with the C/T genotype. Although this analysis is underpowered, these results suggest that the KLW2 G allele and NT213739 C allele lead to epigenetic dysregulation of *Oxtr*, perhaps by impeding the binding of factors that regulate the gene. Further studies with appropriate sample size will be necessary to study this phenomenon. These studies may shed light on mechanisms underlying the common finding in human studies that *OXTR* DNA methylation interacts with genotype at *OXTR* SNP rs53576 in certain psychopathologies [23, 31, 35].

The RNA-sequencing results led to the identification of a novel transcript of *Oxtr*. This transcript, which is homologous to mouse *Oxtr* isoform H (GenBank KU686801.1) [39], originates in the third intron of *Oxtr*. Expression of this transcript is associated with the A allele of KLW2, though further work is necessary to determine if this SNP is functionally related to transcription of this alternative transcript. In animals with a KLW2 A allele, expression of the main transcript and the alternative transcript of *Oxtr* is positively correlated. The function of the novel transcript is unknown. One possibility is that the alternative transcript regulates expression of the main transcript. For example, it has been reported that non-coding RNAs bind DNMT1 to prevent DNA methylation in a gene-specific manner, which in turn increases expression of that gene [51]. If the novel transcript is translated, it would result in a peptide containing most of the seventh transmembrane helix and the C-terminus of OXTR. This peptide could alter the activity of the OXTR protein. Oxtr has been shown to dimerize with type 2 dopamine receptors, though the precise dimerization sites are unknown [52, 53]. It is possible that this OXTR fragment could dimerize with D2Rs, affecting the function of oxytocin–dopamine interactions in the nucleus accumbens. This novel OXTR fragment could also disrupt desensitization of OXTR after oxytocin stimulation, which is dependent on β-arrestin binding to serine triplets in the C-terminus of OXTR [54], by sequestering available β-arrestin. Further studies are necessary to determine the function of this alternative transcript. While this transcript is present in mouse brain, we could not find any evidence of this transcript in human nucleus accumbens using publicly available RNA-seq data from the GTEx project [55]. Further characterization of this transcript in humans will require appropriate, high-quality human brain samples.

Taken together, the results from these experiments offer insight into mechanisms of regulation of *Oxtr* expression in the brain. These results will inform future studies of *OXTR* involvement in human psychopathologies. Further work should focus on the 3′ region of MT2, where DNA methylation is most sensitive to early life experience and most strongly correlates with *OXTR* expression in both prairie vole and human brain. Additional studies in prairie voles focused on the SNPs KLW2 and NT213739 can elucidate genetic by epigenetic interactions in *OXTR* which mediate behavioral outcomes. The identification of a novel transcript of *OXTR*, if also present in humans, has implications for *OXTR* gene regulation, protein function, and behavioral outcomes.

## Conclusions

These results provide strong evidence that DNA methylation in MT2, particularly the 3′ portion, is a better indicator of *OXTR* gene expression than DNA methylation in exon 3 in prairie voles. We also provide evidence that in human temporal cortex, DNA methylation in the 3′ portion of MT2 is associated with *OXTR* gene expression while DNA methylation in other MT2 CpG sites is not. Though associations exist between DNA methylation in *OXTR* exon 3 and neuropsychological outcomes in humans, such results may reflect that DNA methylation in exon 3 is correlated with DNA methylation in MT2. Our findings suggest that future studies of *OXTR* DNA methylation in humans should focus on MT2, particularly CpG sites -934, -924, and -901, as these sites are most related to gene expression in both prairie vole and human brains.

## Methods

### Human brain DNA and RNA samples

Genomic DNA and RNA isolated from the temporal cortex (BA 41/42) was obtained from the Maryland National Institute of Child Health and Human Development Brain Tissue Center and the Harvard Brain Tissue Resource Center (*n* = 11, male, 11-30yrs). Genomic DNA was analyzed for *OXTR* DNA methylation at CpG sites in the MT2 region of the promoter and gene expression was evaluated as previously described (Gregory et al., 2009).

### Animal model

Subjects were laboratory-bred prairie voles (*Microtus ochrogaster*), descendants of a wild-caught stock captured near Champaign, Illinois. Breeding pairs were housed in large polycarbonate cages (44 cm x 22 cm × 16 cm) and same sex offspring pairs were housed in smaller polycarbonate cages (27 cm × 16 cm × 16 cm) after weaning on postnatal day (PND) 20 (date of birth = PND0). Animals were given food (high-fiber Purina rabbit chow) and water ad libitum, cotton nestlets for nesting material in breeding cages, and were maintained on a 14:10 light/dark cycle. All procedures involved in generating tissue for the analysis of DNA methylation and gene expression following handling were reviewed and approved by the IACUC at the University of California, Davis.

### Handling manipulation

Within 24 h of giving birth, breeding pairs underwent a single handling manipulation as previously described [37, 56]. Following this manipulation, parental care is heightened in the MAN1 group compared to the MAN0 group [37]. On PND24 offspring were anesthetized with isoflurane and euthanized via cervical dislocation and rapid decapitation for tissue collection. Brains were extracted and flash frozen in liquid nitrogen and stored at -80 °C. One male and one female from each litter were included in the analysis of *Oxtr* DNA methylation and *Oxtr* expression (MAN0: *n* = 7 per sex; MAN1: *n* = 6 per sex). An additional five sibling pairs were included in the EGA analysis in order to achieve adequate sample size such that the correlation matrix was full rank. These animals did not undergo MAN0 or MAN1 manipulations.

### Sectioning of the nucleus accumbens

Whole brains were stored at -80 °C and equilibrated to -20 °C for two hours prior to sectioning. Following sectioning, nucleus accumbens tissue was placed in a DNAse/RNAse free microcentrifuge tube and flashed frozen with liquid nitrogen. Brain tissue was crushed using a mortar and pestle in preparation for DNA/RNA isolation.

### Identifying conserved MT2 and exonic regions in prairie voles

The structure of *Oxtr* in prairie voles was determined by viewing RNA-sequencing reads near *Oxtr*. The sequence was taken for the whole region where reads were mapped and used to determine conserved regions of the gene. The MT2 region of *OXTR* in humans as identified by Kusui et al*.*, 2001 (GRCh38, chr3:8,769,033–8,769,438) was compared to the *Oxtr* sequence in prairie voles and with the NCBI BLASTn program, and a region in prairie voles was identified as having a highly similar sequence of DNA. To assess the significance of this similarity of the MT2 region between human and prairie vole we used the UVa FASTA server (http://fasta.bioch.virginia.edu/) and PRSS (DNA: DNA) to shuffle the prairie vole sequence 200 times and estimate the statistical significance of the shuffled scores.

### Oxtr DNA methylation analysis

Extraction of DNA from nucleus accumbens tissue was done using the Qiagen AllPrep DNA/RNA Mini Kit (Qiagen, Valencia, CA) following manufacturer instructions. Two hundred nanograms of DNA was subject to bisulfite treatment (Kit MECOV50, Invitrogen, Carlsbad, CA), which allows for the detection of methylated cytosines by sequencing. Twelve nanograms of bisulfite converted DNA was used as template for PCR reactions. PCR was performed using a Pyromark PCR kit (Qiagen, Valencia, CA), and each PCR reaction was amplified in triplicate on three identical PCR machines (S1000 Thermal Cycler, Bio-Rad, Hercules, CA.). PCR primers, PCR cycling conditions, and pyrosequencing primers are reported in Additional file 2. All reactions were completed according to manufacturer instructions, except for the following which all included 3.5 nM MgCl_2_ (provide in the Pyromark PCR kit): MT2, exon 3 Sects. 2–4, exon 3 Sects. 6–8. Standard controls of 0% and 100% methylated DNA, as well as a no DNA control standard, were included for each PCR plate. The median DNA methylation value of the 0% control was 2% (interquartile range: 1%–2%). The median DNA methylation value of the 100% control was 88% (interquartile range: 85%-90%). Following PCR of exon 3 Sect. 1, all samples were run on a 2% agarose gel and a 375 bp product was extracted using the QIAquick Gel Extraction Kit (Qiagen, Valencia, CA) which was subsequently used for pyrosequencing. All samples were randomized for pyrosequencing to account for plate and run variability. Pyrosequencing was performed on a Pyromark Q24 using Pyromark Gold Q24 Reagents (Qiagen, Valencia, CA) per the manufacturer's protocol. Epigenotypes reported are an average of three replicates. The mean deviation from the average ranged from 0.93% (MT2 CpG 7) to 2.29% (MT2 CpG 14).

### Oxtr sequencing and SNP analysis

Ten nanograms of DNA was used as a template for PCR using a Pyromark PCR kit (Qiagen, Valencia, CA). In order to determine the alleles at polymorphic sites, pyrosequencing was completed using unknown base calling on a subset of samples using a Pyromark Q24 with PyroMark Gold Q24 Reagents (Qiagen, Valencia, CA) per the manufacturer protocol. Once alleles were identified, pyrosequencing was performed to measure genotypes of each sample. PCR primers, PCR cycling conditions, and pyrosequencing primers are reported in Additional file 2. The same primers were used for both unknown base calling and genotyping assays. SNPs KLW1 and KLW2 were tested for Hardy–Weinberg equilibrium using the HWExact function in the HardyWeinberg R package [45]. Linkage disequilibrium statistics for KLW1, KLW2, and NT213739 were determined using the LD function in the genetics R package [57].

### Oxtr expression analysis

Extraction of RNA was done using the Qiagen AllPrep DNA/RNA Mini Kit (Qiagen, Valencia, CA) following manufacturer instructions. RNA was processed for cDNA synthesis following the protocol provided in the iScript cDNA Synthesis kit (Bio-Rad, Hercules, CA). Real-time PCR for the *Oxtr* main transcript was conducted using a 7500 Fast Real-Time PCR System (Applied Biosystems) using *Power* SYBR Green (Applied Biosystems No. 4367659). Real-time PCR for the *Oxtr* alternative transcript was conducted using the CFX96 System (Bio-Rad) using *Power* SYBR Green (Applied Biosystems). Real-time PCR for *Pgk1* was completed on both Real-time PCR systems. See Additional file 2 for all RT-PCR primers and cycling conditions. All reactions were run in triplicate (replicate standard deviation was < 0.05) and their specificity verified by melting curve analysis and separation on a 2% agarose gel. Primer performance was evaluated using standard serial dilution and all primer sets performed within acceptable range for efficiency (See Additional file 2). Relative gene expression is presented using the comparative Ct method, 2^−ΔCt^, comparing target expression to *Pgk1* expression measured on the same real-time PCR system. *Pgk1* was chosen as a reference based on data in mouse brain showing its reliability across brain regions and developmental time points [58].

### RNA-sequencing and alignment

To identify the structure of *Oxtr* in prairie voles, we performed transcriptome analysis using RNA-sequencing on a single RNA sample derived from the nucleus accumbens of a female MAN1 vole in our cohort. RNA quality was assessed using an Agilent Tape Station. The RIN score was 9.0. The library was generated from 500 ng RNA using the NEBNext Ultra Directional RNA Library Prep Kit with mRNA magnetic isolation. The UVA Genome Analysis and Technology Core performed paired-end sequencing (2 X 75 bp paired-end run) on the Illumina NextSeq 500 platform. A total of 125 million reads were generated. The raw sequencing data was subjected to preprocessing steps of adapter removal and quality-based trimming using TrimGalore [59] with the removal of auto-detected Illumina adapters and trimming of low-quality ends up to a threshold of Q20. Reads that became shorter than 35 bp due to either adapter removal or quality trimming were discarded. Quality control was completed with MultiQC [60]. Novel transcript identification and quantification was performed using a standard analysis approach outlined elsewhere [61]. Briefly, sequencing data were aligned to the *Microtus ochrogaster* genome using a splice-aware aligner STAR2 [62], followed by assembly and quantification using Stringtie [63]. Alignments were viewed in the Integrated Genomics Viewer [64].

### Statistical analysis

Statistical computing and graphics were generated with R statistical software [65]. Graphics were generated using the ggplot2 and ggpubr packages unless otherwise stated [66, 67]. For each analysis, *p* < 0.05 was regarded as statistically significant with respect to multiple comparison procedures as appropriate. The effect of handling on DNA methylation in MT2 and exon 3 was determined using a mixed effects model with fixed effect of handling condition and random effects of intercepts for individual and CpG site using the lme4 package [68]. The type II Wald F test in the car package was used to determine significance of fixed effects [69]. The effect of handling condition on DNA methylation at individual CpG sites was determined using t tests followed by Bonferroni correction. In both humans and in prairie voles, Spearman’s rank correlation was used to determine the relationship between DNA methylation and gene expression and uncorrected *p* values are reported because the distribution of DNA methylation at some CpG sites in human MT2, prairie vole MT2, and prairie vole exon 3 are non-normal. For visualization purposes, we plotted trend lines from linear regression models predicting DNA methylation from expression. The community structure of the MT2 region was determined using exploratory graph analysis (EGA) implemented in the EGAnet package [70]. We estimated the EGA network using the Triangulated Maximally Filtered Graph (TMFG) model [71] as opposed to the graphical LASSO (GLASSO) model, because the GLASSO procedure typically requires n (sample size) >  > p (CpG sites) to accurately compute partial correlations for each site. TMFG skirts this issue by modeling the zero-order correlation matrix, rather than the partial correlation matrix. The effect of handling condition on standardized network community score was determined using a two-way community x handling condition ANOVA, followed by t tests within each community and Bonferroni correction. Spearman’s rho was used to determine the relationship between standardized network community score and *Oxtr* expression because the distribution of standardized network community scores is non-normal. The impact of *Oxtr* polymorphisms KLW1 and KLW2 (both A/G SNPs) on gene expression was determined using a t test comparing A carriers (A/A and A/G) to G/G homozygotes. The impact of sex, handling condition, and *Oxtr* polymorphisms KLW1, KLW2, and NT213739 on expression of the alternative transcript were determined using the Wilcoxon Rank Sum test as expression of the alternative transcript was non-normal.

## Supplementary Information

**Additional file 1**. Supplementary figures and tables.

**Additional file 2**. Primers and PCR conditions for DNA methylation analysis and RT-PCR.

## Data Availability

RNA-sequencing data from the prairie vole nucleus accumbens are available upon reasonable request. All other data generated and analyzed in this study are present in the published article.
