# Supplementary material for: Genetic, epigenetic, and environmental factors controlling oxytocin receptor gene expression
Source: Clin Epigenetics. 2021 Jan 30;13:23. doi: 10.1186/s13148-021-01017-5 (PMC7847178; doi:10.1186/s13148-021-01017-5)
Supplement: Supplementary file 1 — Additional file 1. Supplementary figures and tables. [file 13148_2021_1017_MOESM1_ESM.docx]

Figure S1. Alignment of conserved MT2 ad exon 3 regions in prairie voles and humans. A) Alignment of MT2 region in prairie voles (micOch1.0 JH996431.1:26,357,582-26,357,959) and humans (GRCh38, chr3: 8,769,034-8,769,438). In both prairie voles and humans, the minus (coding) strand is shown. Prairie vole CpGs are in bold and numbered 1 through 24, with the exception of previously studied CpG sites -934_1, -934_2, -924, and -901, which are named according to homology with human CpG sites. Human CpGs are also in bold; select sites which have been previously studied or are directly conserved in prairie voles are named according to distance from the translation start site (1). B) Alignment of exon 3 region in prairie voles (micOch1.0 JH996431.1:26,356,019-26,356,593) and humans (GRCh38 chr3: 8,767,386-8,767,963). In both prairie voles and humans, the minus (coding) strand is shown. CpGs in both humans and prairie voles are in bold; prairie vole CpGs are numbered 1 through 42.

Figure S2. Dimensionality reduction of MT2 using Exploratory Graph Analysis. A) Correlation matrix for DNA methylation in MT2 in prairie vole nucleus accumbens. B) Scree plot indicating that DNA methylation in MT2 can be represented using 3 linear combinations. C) Adjacency matrix indicating remaining correlations after dimension reduction displaying correlations of DNA methylation at CpG sites with connections in the EGA network.


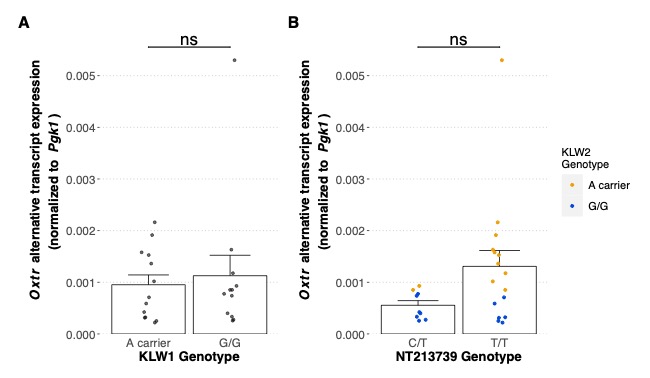


Figure S3. Expression of the *Oxtr* alternative transcript is not impacted by KLW1 or NT213739 genotype. A) There is no significant difference in *Oxtr* alternative transcript expression levels between KLW1 genotypes (A carrier: n=14, G/G: n=12; Wilcoxon rank sum test, W=77, p=0.98). B) There is so significant difference in *Oxtr* alternative transcript expression levels between NT213739 genotypes (C/T: n=9, T/T: n=17; Wilcoxon rank sum test, W=42, p=0.1). The data points are colored according to KLW2 genotype to show that the observed increased present in NT213739 T/T animals is because of increased numbers of KLW2 A carriers.

**Table S1. Correlation of MT2 CpG Sites with *Oxtr* gene expression in prairie vole brain.**

| MT2 CpG Site | Genomic Coordinate (bp)  (michOch1.0) | ρ | p value |
| --- | --- | --- | --- |
| 1 | JH996431.1: 26,357,956 | -0.282608696 | 0.180369104 |
| 2 | JH996431.1: 26,357,952 | 0.165217391 | 0.438653492 |
| 3 | JH996431.1: 26,357,905 | -0.145248971 | 0.498285055 |
| 4 | JH996431.1: 26,357,892 | -0.273162393 | 0.176435702 |
| 5 | JH996431.1: 26,357,881 | -0.228120797 | 0.272750625 |
| 6 | JH996431.1: 26,357,877 | -0.210434783 | 0.322103767 |
| 7 | JH996431.1: 26,357,872 | -0.297749573 | 0.148302135 |
| 8 | JH996431.1: 26,357,869 | -0.235429895 | 0.257256834 |
| 9 | JH996431.1: 26,357,848 | -0.376923077 | 0.064109728 |
| 10 | JH996431.1: 26,357,839 | -0.355897436 | 0.075012607 |
| 11 | JH996431.1: 26,357,837 | -0.272307692 | 0.187272965 |
| 12 | JH996431.1: 26,357,819 | **-0.455726496** | **0.020292825** |
| 13 | JH996431.1: 26,357,814 | -0.273162393 | 0.176435702 |
| 14 | JH996431.1: 26,357,794 | -0.347692308 | 0.082347948 |
| 15 | JH996431.1: 26,357,743 | -0.316923077 | 0.114856522 |
| 16 | JH996431.1: 26,357,738 | -0.238974359 | 0.238642685 |
| 17 | JH996431.1: 26,357,701 | **-0.547350427** | **0.004354648** |
| 18 | JH996431.1: 26,357,671 | **-0.435897436** | **0.027038443** |
| -934_1 | JH996431.1: 26,357,648 | -0.293675214 | 0.145172394 |
| -934_2 | JH996431.1: 26,357,642 | **-0.508376068** | **0.00877823** |
| -924 | JH996431.1: 26,357,628 | **-0.458461538** | **0.019482501** |
| -901 | JH996431.1: 26,357,607 | **-0.454358974** | **0.020708305** |
| 23 | JH996431.1: 26,357,603 | **-0.44957265** | **0.022218149** |
| 24 | JH996431.1: 26,357,600 | **-0.413076923** | **0.041186079** |

Correlation between DNA methylation in MT2 and gene expression was measured using Spearman’s rho. Bolded sites indicate significant correlation of DNA methylation and *Oxtr* expression. p-values are not adjusted for multiple comparisons. CpG sites in MT2 are named according to Figure 1C. Genomic coordinates are provided using genome build michOch1.0 ([**GCA_000317375.1**](https://www.ncbi.nlm.nih.gov/assembly/GCA_000317375.1)).

**Table S2. Correlation of MT2 CpG Sites with *OXTR* gene expression in human brain (BA41/42).**

| MT2 CpG Site | Genomic coordinate (bp)  (GRCh38.p12 /hg38) | ρ | p value |
| --- | --- | --- | --- |
| -1155 | Chr3: 8,769,343 | NA | NA |
| -1137 | Chr3: 8,769,325 | NA | NA |
| -1132 | Chr3: 8,769,320 | 0.5 | 0.1173068 |
| -1126 | Chr3: 8,769,314 | -0.3 | 0.37008312 |
| -1122 | Chr3: 8,769,310 | 0.5 | 0.1173068 |
| -1120 | Chr3: 8,769,308 | NA | NA |
| -1109 | Chr3: 8,769,297 | -0.5597585 | 0.07334733 |
| -1101 | Chr3: 8,769,289 | 0.3 | 0.37008312 |
| -1098 | Chr3: 8,769,286 | -0.094388 | 0.78251392 |
| -1085 | Chr3: 8,769,273 | 0.07515581 | 0.82616973 |
| -1063 | Chr3: 8,769,251 | 0.57981188 | 0.06152632 |
| -1057 | Chr3: 8,769,245 | -0.175292 | 0.60618047 |
| -1051 | Chr3: 8,769,239 | 0.31013194 | 0.35332506 |
| -1017 | Chr3: 8,769,205 | 0.1 | 0.769875 |
| -1002 | Chr3: 8,769,190 | 0.5 | 0.1173068 |
| -990 | Chr3: 8,769,178 | -0.4 | 0.22286835 |
| -983 | Chr3: 8,769,171 | -0.363756 | 0.27146808 |
| -959 | Chr3: 8,769,147 | -0.1284458 | 0.70663663 |
| -934 | Chr3: 8,769,122 | **-0.7818182** | **0.00701211** |
| -924 | Chr3: 8,769,112 | **-0.6833731** | **0.02043986** |
| -901 | Chr3: 8,769,089 | **-0.6560381** | **0.02837692** |
| -860 | Chr3: 8,769,048 | -0.2186794 | 0.518272 |

Correlation between DNA methylation in MT2 and gene expression was measured using Spearman’s rho. Bolded sites indicate significant correlation of DNA methylation and *Oxtr* expression. p-values are not adjusted for multiple comparisons. CpG sites in MT2 are named according to distance from translation start site, as was done in Gregory, Connelly et al (1). Genomic coordinates are provided using Genome Reference Consortium Human GRCh38.p12 (GCA_000001405.27). At some CpG sites, there was no variation in DNA methylation, as all subjects had 0% methylation. For these sites, Spearman’s rho could not be calculated.

**Table S3. Correlation of Exon 3 CpG Sites with *Oxtr* gene expression.**

| Site | Genomic Coordinates (bp)  (michOch1.0) | ρ | p value |
| --- | --- | --- | --- |
| 1 | JH996431.1: 26,356,568 | -0.21983 | 0.279245 |
| 2 | JH996431.1: 26,356,533 | -0.27521 | 0.173111 |
| 3 | JH996431.1: 26,356,507 | -0.24444 | 0.227812 |
| 4 | JH996431.1: 26,356,499 | -0.31897 | 0.112429 |
| 5 | JH996431.1: 26,356,493 | -0.19863 | 0.329126 |
| 6 | JH996431.1: 26,356,480 | -0.2294 | 0.258417 |
| 7 | JH996431.1: 26,356,445 | -0.22667 | 0.26426 |
| 8 | JH996431.1: 26,356,416 | -0.18496 | 0.364051 |
| 9 | JH996431.1: 26,356,412 | -0.09265 | 0.6515 |
| 10 | JH996431.1: 26,356,408 | -0.08581 | 0.675853 |
| 11 | JH996431.1: 26,356,386 | -0.18222 | 0.371291 |
| 12 | JH996431.1: 26,356,381 | -0.19179 | 0.346322 |
| 13 | JH996431.1: 26,356,372 | -0.18974 | 0.351584 |
| 14 | JH996431.1: 26,356,369 | -0.15077 | 0.460516 |
| 15 | JH996431.1: 26,356,366 | -0.19658 | 0.334229 |
| 16 | JH996431.1: 26,356,361 | -0.15832 | 0.439846 |
| 17 | JH996431.1: 26,356,357 | -0.1665 | 0.414551 |
| 18 | JH996431.1: 26,356,350 | -0.12479 | 0.542051 |
| 19 | JH996431.1: 26,356,341 | -0.11521 | 0.573724 |
| 20 | JH996431.1: 26,356,313 | -0.24103 | 0.234541 |
| 21 | JH996431.1: 26,356,311 | -0.17197 | 0.399192 |
| 22 | JH996431.1: 26,356,308 | -0.32923 | 0.100864 |
| 23 | JH996431.1: 26,356,285 | -0.32513 | 0.105377 |
| 24 | JH996431.1: 26,356,283 | -0.25949 | 0.199765 |
| 25 | JH996431.1: 26,356,275 | -0.20342 | 0.31741 |
| 26 | JH996431.1: 26,356,271 | -0.31522 | 0.142866 |
| 27 | JH996431.1: 26,356,212 | -0.20957 | 0.302733 |
| 28 | JH996431.1: 26,356,203 | -0.20615 | 0.310833 |
| 29 | JH996431.1: 26,356,196 | -0.16991 | 0.404913 |
| 30 | JH996431.1: 26,356,166 | -0.15419 | 0.450299 |
| 31 | JH996431.1: 26,356,123 | -0.21162 | 0.297938 |
| 32 | JH996431.1: 26,356,113 | -0.27726 | 0.16983 |
| 33 | JH996431.1: 26,356,107 | -0.2588 | 0.200985 |
| 34 | JH996431.1: 26,356,104 | -0.12068 | 0.555523 |
| 35 | JH996431.1: 26,356,101 | -0.1159 | 0.571434 |
| 36 | **JH996431.1: 26,356,097** | **-0.39077** | **0.049325** |
| 37 | JH996431.1: 26,356,073 | -0.1453 | 0.477117 |
| 38 | JH996431.1: 26,356,060 | -0.13641 | 0.504746 |
| 39 | JH996431.1: 26,356,056 | -0.21641 | 0.286939 |
| 40 | JH996431.1: 26,356,053 | 0.063932 | 0.755875 |
| 41 | JH996431.1: 26,356,045 | 0.026325 | 0.898975 |
| 42 | JH996431.1: 26,356,034 | -0.05299 | 0.796906 |

Correlation between DNA methylation in exon 3 and gene expression was measured using Spearman’s rho. Bolded sites indicate significant correlation of DNA methylation and *Oxtr* expression. p-values are not adjusted for multiple comparisons. CpG sites in exon 3 are named according to Figure 1D. Genomic coordinates are provided using genome build michOch1.0 (**Assembly accession ID:** [**GCA_000317375.1**](https://www.ncbi.nlm.nih.gov/assembly/GCA_000317375.1)).

**References**

1. S. G. Gregory, J. J. Connelly, *et al.*, Genomic and epigenetic evidence for oxytocin receptor deficiency in autism. *BMC Med.* **7** (2009).
